# Heterochromatin: A Rapidly Evolving Species Barrier

**DOI:** 10.1371/journal.pbio.1000233

**Published:** 2009-10-27

**Authors:** Stacie E. Hughes, R. Scott Hawley

**Affiliations:** 1 Stowers Institute for Medical Research, Kansas City, Missouri, United States of America; 2 Department of Physiology, University of Kansas Medical Center, Kansas City, Kansas, United States of America

## Abstract

Recent work has shown that changes in the sequence composition of heterochromatin, or in the factors that maintain that heterochromatin, may play an important role in speciation.

Nearly 100 years ago, biologists divided regions of chromosomes into two types, euchromatin and heterochromatin, on the basis of their appearance (reviewed in [1]). The initial classification of DNA was based on the observation that euchromatic regions changed their degree of condensation during the cell division cycle, whereas heterochromatic regions remained highly condensed throughout the majority of the cell cycle. Although the biological significance of heterochromatin remained obscure for many years, it is now apparent that heterochromatin plays a number of biological roles, including a recently identified role in speciation. In addition to differences in the timing of chromosome condensation, numerous other differences have been identified between euchromatin and heterochromatin. Euchromatin is enriched with unique coding sequences, and the genes within the euchromatin are typically actively transcribed. Heterochromatin, on the other hand, is considered to be gene poor, being primarily composed of arrays of highly repetitive simple sequences, such as satellite sequences and/or transposable elements. Heterochromatin is enriched at the centromeres (see Figure 1) and telomeres of chromosomes.

**Figure 1:**
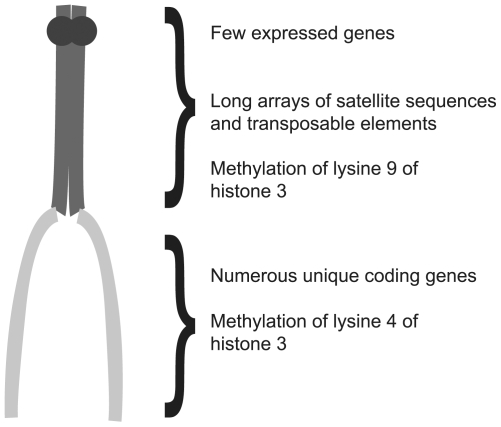
DNA can be divided into euchromatin and heterochromatin. Shown is a representative acrocentric chromosome containing both condensed heterochromatic (dark gray) and less condensed euchromatic regions (light gray). Beside each region are characteristics typical for each type of chromatin.

A number of chromatin modifications are associated specifically with either heterochromatin or euchromatin, such as specific methylation patterns on the histones. Proteins involved in creating the histone methylation patterns associated with heterochromatin, as well as proteins, such as heterochromatin protein 1 (HP1) that are involved in heterochromatin formation and gene silencing, are preferentially found localized to heterochromatic DNA. Finally, heterochromatin appears to be rapidly evolving, so that the sequence composition of heterochromatic regions from even closely related species is often distinct. The scarcity of genes in heterochromatin, as well as its rapid evolution, led many 20th century scientists to view heterochromatin as no more than “junk” DNA with little biological importance, other than comprising, and perhaps protecting, the centromeres and telomeres.

The fruit fly *Drosophila melanogaster* and its close relatives have been popular models for studying the nature and formation of heterochromatin (reviewed in [1]). In these species, euchromatin can easily be distinguished from pericentric heterochromatin, which surrounds the centromeres of mitotic chromosomes, by cytology owing to the differences in the condensation of the two types of DNA. Moreover, heterochromatic regions fail to replicate in the polytene chromosomes, which are highly replicated chromosomes that remain tightly associated in the larval salivary glands. This allows a reasonably precise demarcation of the junction between euchromatin and heterochromatin. Additionally, *D. melanogaster* is a highly tractable system for conducting genetic screens and for performing forward genetic manipulations. Because both heterochromatin and euchromatin are easily genetically dissected in *D. melanogaster*, the biology of heterochromatin has been intensely studied using this organism.

Indeed, work in *D. melanogaster* has begun to challenge the view of heterochromatin as “junk” DNA and demonstrated that heterochromatin plays a number of important cellular functions. The first indications of a “function” for heterochromatin came from studies of the multiple roles of heterochromatin in mediating recombination during meiosis [2],[3]. Perhaps more critically, placing normally euchromatic genes near heterochromatin causes the variable silencing of these genes, an effect known as position-effect variegation, or PEV (reviewed in [1]). This silencing effect and the fact that some genes must be in heterochromatin in order to be properly transcribed suggest that heterochromatin may play a critical role in the global control of gene regulation [4]–[6].

Moreover, experiments by Karpen, Dernburg, Hawley, and their collaborators have demonstrated that pairing of heterochromatic regions is required for the proper segregation of chromosomes that fail to undergo recombination during female meiosis [7]–[9]. Subsequent work demonstrated that chromosomes that fail to undergo recombination (the *X* and *4^th^*) are connected by heterochromatic threads during prometaphase I in oocytes and that these threads are likely part of the mechanism by which heterochromatin facilitates nonrecombinant chromosome segregation [10].

Evidence for threads connecting chromosomes during meiosis in the sperm of both *D. melanogaster* and crane flies suggest a conserved function for thread-like structures in the segregation of chromosomes during meiosis [11],[12]. Although it has not yet been determined whether these threads are composed of heterochromatin, the repetitive intergenic spacer region of rDNA, which resides in heterochromatin, is required for the proper pairing of the *Y* chromosome with the *X* chromosome during male meiosis in *D. melanogaster* [13]. Finally, similar types of threads have been observed emanating from the heterochromatic centromere regions of chromosomes during mitosis in mammalian cells, suggesting that heterochromatic threads play an important role in chromosome segregation during mitosis as well [14],[15].

Given the critical functions of heterochromatic sequences in both meiosis and mitosis and its rapid change in sequence throughout evolution, it might not be surprising if differences in either heterochromatic sequences or the proteins that maintain them might indeed play a role in species isolation [16]. Mating of related species sometimes leads to the death or sterility of one or both sexes of progeny, which is known as hybrid incompatibility. Although hybrid incompatibility has been studied for decades, there have been only a few insights into the molecular mechanisms that underlie it, and in those cases known for the *Drosophila* species, the hybrid incompatibility has involved protein-coding genes [17],[18]. However, in this issue of *PLoS Biology* Ferree and Barbash demonstrate that the rapid divergence of heterochromatin also plays an important role in maintaining the reproductive isolation of *D. melanogaster* from the sister species *Drosophila simulans* [16]. The cross between *D. simulans* females and *D. melanogaster* males is unusual in that male offspring are viable but females die during embyronic development [19]. (Typically in cases of hybrid incompatibility the heterogametic males are the affected sex if only one sex is sterile or lethal.)

Ferree and Barbash found the lethality in hybrid female embryos resulted from failures during mitotic divisions 10–13 [16]. In these females, chromosomal regions frequently appeared highly stretched and lagged behind the other chromosomes during anaphase of these mitotic divisions [16]. The lagging DNA failed to become properly separated from its sister during mitosis, leading to improper chromosome segregation, aberrant mitotic divisions, and, ultimately, the death of female embryos.

Using fluorescent in situ hybridization, the authors determined that the lagging chromatin was primarily composed of the heterochromatic and highly repetitive 359-bp repeat on the *X* chromosome from the *D. melanogaster* father [16]. This particular heterochromatic repeat type has a different sequence composition and is much less abundant in *D. simulans*. The 359-bp repeat-containing region on the *D. melanogaster X* chromosome also overlaps the *Zhr* locus, a genetic region that was identified because its deletion from the *D. melanogaster X* chromosome allowed *D. melanogaster* males to produce viable hybrid daughters when crossed to *D. simulans* females [19].

The discovery that lethality in hybrid females results from a failure to maintain the integrity of a heterochromatic region of the *D. melanogaster X* chromosome containing the 359-bp repeat sequence suggests an intriguing possibility, namely, that the chromosome lagging and lethality of the *D. melanogaster X* chromosomal heterochromatin in female hybrids occurs because the *D. simulans* mother fails to provide a protein or RNA molecule required for proper maintenance and or separation during mitosis of the 359-bp repeat region provided by the *D. melanogaster* father (see Figure 2). One might even imagine that the failure in mitotic segregation observed here reflects a failure to properly resolve the heterochromatic threads observed to connect the pericentromeric heterochromatin in both mitotic and meiotic cells (see above).

**Figure 2:**
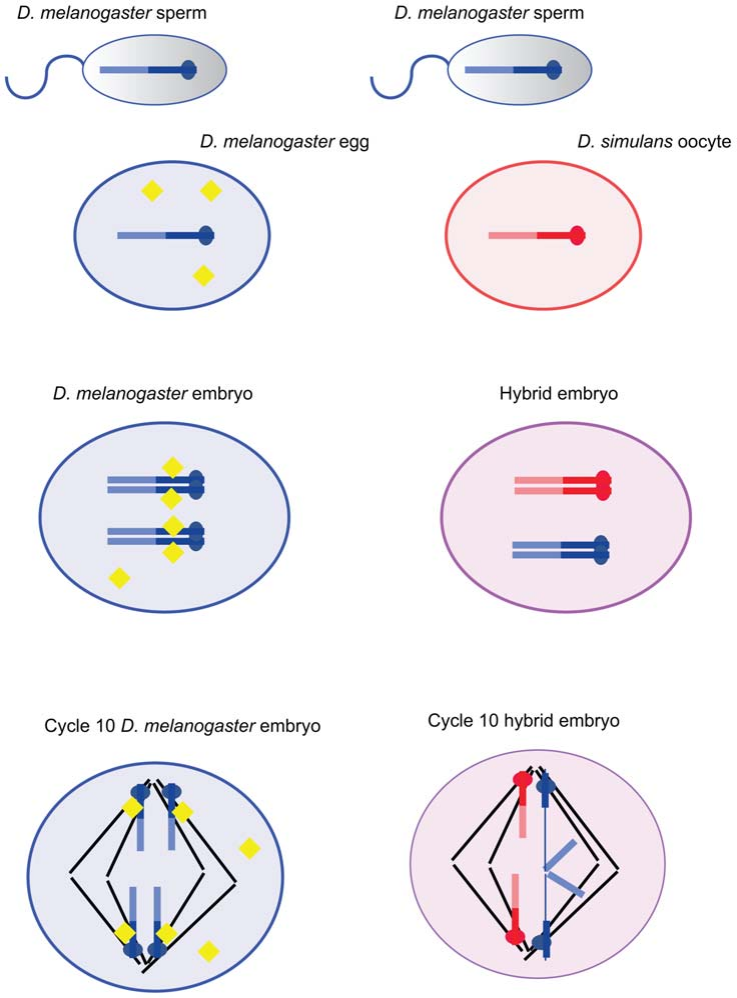
A model of how the mishandling of a specific heterochromatic region might cause lethality in female hybrid embryos. This model is based on the results of the article published by Ferree and Barbash in this issue of *PLoS Biology* [16]. A cross between *D. melanogaster* males and *D. simulans* females, which results in hybrid females that die early in embryogenesis, is shown on the right. The drawing on the left depicts a cross between *D. melanogaster* males to *D. melanogaster* females for comparison. For simplicity, only the *X* chromosomes are shown and the heterochromatic region is specified with a darker color. In both crosses, the fusion of the sperm and egg results in zygotes carrying a pair of *X* chromosomes. The cross with the *D. melanogaster* females leads to normal chromosome segregation during anaphase of mitotic divisions 10–13 in female embryos. In the cross with *D. simulans* females mitosis fails to be completed normally in the hybrid female embryos. While the maternal *X* chromosomes segregate normally towards opposite spindle poles, the segregating centromeres of the paternally derived *X* chromosomes are connected by a bridge of chromatin. This bridge, which is heterochromatic and comprised of a region rich in the 359-bp repeat, causes improper segregation of the sister chromatids of the *X* chromosome, an event that eventually leads to aberrant mitotic divisions and ultimately the death of female hybrid embryos. The lagging or bridging of the 359-bp region is likely due to an absence of a maternally loaded factor in the *D. simulans* egg (shown as yellow diamonds in the *D. melanogaster* egg). We imagine that this factor might be involved in the resolution of heterochromatic threads that have been shown to connect the pericentromeric regions of both mitotic and meiotic chromosomes in normal cells (see text for a description). The absence of this factor prevents the proper formation or maintenance of chromatin structure in the 359-bp repeat region in female hybrid embryos.

The authors suspected that *D. simulans* lacks a factor required to either maintain heterochromatic stability or to resolve heterochromatic linkages in *D. melanogaster X* chromosomal heterochromatin during embryonic mitotic divisions. Indeed, they found that topoisomerase II, an enzyme required for proper mitosis, showed aberrant localization to the lagging DNA in hybrid embryos. Further work will be required to determine if other proteins or RNA molecules are absent or aberrantly localized from the *D. simulans* maternal cytoplasm, and if their absence is sufficient to cause the defects in the 359-bp repeat region in hybrid female embryos.

While this is the first example of a heterochromatic sequence causing hybrid incompatibility, other instances will likely be found in nature. Indeed, we cannot help but note a parallel between this example of hybrid inviability and a genetic phenomenon known as segregation distortion, which has also been well studied in *D. melanogaster* [20]. In this system a novel mutant known as *SD*, which is located in the euchromatin of Chromosome *2*, prevents the meiotic transmission of homologous *2^nd^* chromosomes carrying high copy numbers of a heterochromatic element known as *Responder* (*Rsp*) [21]. The genetic basis of this phenomenon, which causes improper condensation and function of *Rsp*-bearing spermatids, is well understood, and a full molecular understanding of this process is within reach. While segregation distortion is indeed an example of “meiotic drive,” and not a species isolation mechanism, it bears mention here because it illustrates a second case in which a mutant in one strain impairs the function of a heterochromatic element in another; thus illustrating the mechanisms of “heterochromatic incompatibility” may be more common than one might have expected.

As heterochromatin rapidly changes, the mechanisms that maintain it may well diverge as populations become isolated by various mechanisms. If those mechanisms change in such a way that the heterochromatin of population A can no longer be maintained by the maintenance proteins in population B, then the heterochromatin itself becomes a barrier between those populations as speciation proceeds. Many more questions await investigation, both in terms of the system of hybrid inviability described above and in terms of assessing the degree to which the safe-guarding of heterochromatic integrity underlies other examples of speciation. But one thing is clear: if any part of heterochromatin is indeed “junk,” then it is “junk” that both needs to be taken good care of and “junk” that sets one species apart from its neighbors.
